# A non‐invasive approach to measuring body dimensions of wildlife with camera traps: A felid field trial

**DOI:** 10.1002/ece3.11612

**Published:** 2024-07-01

**Authors:** Alexandra J. Paton, Barry W. Brook, Jessie C. Buettel

**Affiliations:** ^1^ School of Natural Sciences University of Tasmania Hobart Tasmania Australia; ^2^ ARC Centre of Excellence for Australian Biodiversity and Heritage (CABAH) University of Tasmania Hobart Tasmania Australia

**Keywords:** body size, *Felis catus*, invasive species, non‐intrusive, trail camera

## Abstract

Dimensions of body size are an important measurement in animal ecology, although they can be difficult to obtain due to the effort and cost associated with the invasive nature of these measurements. We avoid these limitations by using camera trap images to derive dimensions of animal size. To obtain measurements of object dimensions using this method, the size of the object in pixels, the focal length of the camera, and the distance to that object must be known. We describe a novel approach of obtaining the distance to the object through the creation of a portable distance marker, which, when photographed, creates a “reference image” to determine the position of the animal within an image. This method allows for the retrospective analysis of existing datasets and eliminates the need for permanent in‐field distance markers. We tested the accuracy of this methodology under controlled conditions with objects of known size resembling *Felis catus*, our study species, validating the legitimacy of our method of size estimation. We then apply our method to measure feral cat body size using images collected in Tasmania, Australia. The precision of our methodology was evaluated by comparing size estimates across individual cats, revealing consistent and reliable results. The average height (front paw to shoulder) of the feral cats sampled was 25.25 cm (CI = 24.4, 26.1) and the average length (base of tail to nose) was 47.48 cm (CI = 46.0, 48.9), suggesting wild feral cats in our study area are no larger than their domestic counterparts. Given the success of its application within our study, we call for further trails with this method across a variety of species.

## Graphical abstract

Camera‐traps can provide a non‐invasive alternative to measuring dimensions of animal body size. We describe a novel approach of obtaining the distance to an animal from a camera‐trap required for this method through the creation of a portable distance marker and subsequent “reference image.” We tested the accuracy of this methodology under controlled conditions with objects of known size resembling *Felis catus*, our study species, validating the legitimacy of our method of size estimation and apply our method to feral cat images collected in Tasmania, Australia. We compared size estimates across individual cats, revealing consistent and reliable results. The average height (front paw to shoulder) of the feral cats sampled was 25.25 cm (CI = 24.4, 26.1) and length (base of tail to nose) was 47.48 cm (CI = 46.0, 48.9), suggesting wild feral cats in our study area are no larger than their domestic counterparts.

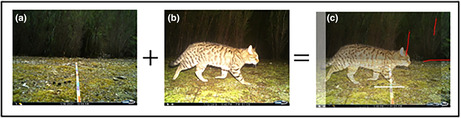

## 1 INTRODUCTION

Body size is a frequently measured attribute that can provide information on the sex, diet, body condition, and potential predators of an animal (Cumming & Cumming, 2003; Gittleman & Valkenburgh, 1997). The most common methodologies used to measure body dimensions (hereafter, “size”) involve opportunistic measurements being taken during live trapping, tranquilizing, or killing of an animal (Richard‐Hansen et al., 1999). These methods allow for a range of biometric measurements, including weight, tissue and blood samples, and the specific dimensions of size. However, this direct contact with wild animals may alter their behavior, cause high levels of stress, and potentially result in injury or death (Zemanova, 2020). Live captures are also laborious and financially costly, meaning an intense sampling effort is required to obtain sufficient sample sizes (De Bondi et al., 2010; Mills et al., 2016). In contrast, camera trapping offers a cost effective, non‐invasive monitoring method that provides information on the presence, activity, and potential density of a target species (Kays et al., 2020), although there are limited opportunities to gain qualitative biometric information through this approach. Considering the visual nature of the data provided by camera traps, there is the potential to opportunistically measure animal size using this non‐invasive methodology.

Past research has explored estimating animal size directly from camera trap images. A relatively simple method is described by Tarugara et al. (2019). They deployed carcasses atop fallen trees, and secured steel pegs 20 cm apart on the underside of these logs, so that as leopards (*Panthera pardus*) climbed the trees to retrieve the carcasses, their body dimensions could be estimated from this permanent scale. This methodology worked because the animal could be continuously captured in the same position and distance from the camera, allowing for the scale to provide an accurate reference point. However, such effective designs are not always possible, as animals may vary in their distance from the camera trap, limiting the information a permanent scale can provide.

Leorna et al. (2022) demonstrate the utility of the pinhole camera approach in circumstances where a permanent scale cannot be used. They employed the pinhole method to provide accurate measurements of reindeer (*Rangifer tarandus*) body dimensions from camera trap images. To use the method, one must have measurements of the body dimension in pixels, the focal length of the camera, and the distance of the animal to the camera trap (Johanns et al., 2022; Leorna et al., 2022). Measuring the distance of an animal to the camera trap from within camera trap images can prove problematic. One approach has been to place distance markers at regular intervals in the camera's field of view for the duration of the monitoring period (Corlatti et al., 2020; Hofmeester et al., 2017). However, these markers are conspicuous and could increase the risk of theft or alter the behavior of the study species (Corlatti et al., 2020). These markers also tend to bin distances at quite wide intervals (i.e., 1‐ to 2‐meter intervals) (Corlatti et al., 2020; Leorna et al., 2022). A potential alternative is to use a laser rangefinder to derive distance (Leorna et al., 2022), although this option is expensive (~$500 USD per unit), therefore limiting the number of potential stations that can be deployed and inflating the consequences of theft and vandalization.

In this study, we describe a cost‐effective, low‐effort, and inconspicuous method for estimating size utilizing the pinhole camera approach, allowing for reliable measurements whilst keeping the risk of disturbance and theft low. Our method can be implemented in pre‐existing camera trap surveys and does not require permanent distance markers. Our approach can also be retroactively applied to historical data. We demonstrate the utility of this method with feral cats, an ecologically damaging, trap‐shy invasive predator in Australia. In estimating their size, we also seek to uncover whether feral cats in Tasmania (our study region) are larger than domestics, addressing the common anecdotal reports of “giant cats” in the wilds of Australia (Menkhorst & Morison, 2012).

## 2 METHODS

### 2.1 Estimating size from a camera trap image

The following steps are required for our approach: (i) source or calibrate the focal length of the camera trap; (ii) create a portable reference marker; (iii) take a reference image of the marker at each camera trap location; (iv) overlay the image with animal images from the same site in photo‐editing software; (v) measure desired dimensions of the animal, in pixels, as well as the distance to the animal; and (vi) convert these measurements from pixels to meters. Each of these steps are described in detail below, with illustrations.

### 2.2 Information required to calculate size

The method described within this paper employs the pinhole camera approach, which illustrates the relationship between a two‐dimensional image and a three‐dimensional scene as described by this equation:

$$\frac{S_{i}}{d_{i}}=\frac{S_{o}}{d_{o}}$$

where *S*_*i*_ is the size of the object on the image in pixels, d_i_ is the distance of the camera sensor to the aperture (i.e., focal length expressed in pixels), *S*_o_ is the physical size of the object (in meters), and d_o_ is the physical distance to the object in meters from the camera (Leorna et al., 2022).

Three pieces of information are needed to rearrange this equation to obtain the physical size of an object/animal from a camera trap image. These include the camera trap's focal length (*d*_*i*_), measurements in pixels of the animal's dimensions, and the distance of the animal to the camera trap (*d*_o_). Focal length can occasionally be sourced from a camera trap manufacturer, but can more reliably be obtained by following the methods of Megalingam et al. (2016), which describe a calibration procedure to derive focal length in pixels. The focal length is a static specification that will not change between calculations, provided that the researcher is using the same camera model throughout their monitoring. In contrast, the pixel measurements of an animal's dimensions and the distance of the animal to the camera trap will be different for each photograph and camera trapping site. As such, a distance marker is required to provide a reference point for the distance of an animal to the camera trap.

### 2.3 Distance marker and in‐field protocol

**FIGURE 1:**
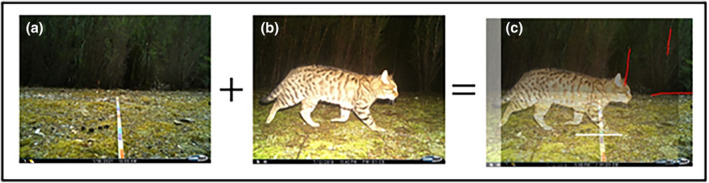
An example of the field photo taken of the measuring tape with 10 cm color blocks out to 230 cm (panel a), a suitable image of a cat close enough to the camera for analysis (panel b), and how the images are superimposed, aligned and sanity checked to ensure the tape measure is in an accurate position to read distance of the cat to the camera. The red lines in panel (c) show reference points in the background that were traced to ensure accurate overlay, and the white line indicates the distance of the cat from the camera trap on the distance marker.

Implementing this method requires researchers to create a distance marker that can be readily taken into the field. This distance marker needs to maintain a straight line from the camera trap and provide visible indicators of distance at regular intervals to be readily discerned from the camera trap images. The design of the distance marker is flexible, and largely dependent on the resources and requirements of the researcher (and camera model). For our study, we created a portable distance marker using a tape measure marked with different colors every 10 cm, to total length of 230 cm (Figure 1). This length was determined as the longest distance at which the colors on the tape were still reliably distinguishable within a camera trap image taken by a Cuddeback X‐Change Colour Model 1279 with 20‐megapixel resolution (the standard device we used throughout this work). This maximum distance may vary for other cameras, depending on their specifications.

A reference image of the distance marker must be taken at each camera trap site (e.g., Figure 1, panel a). This image will later be overlain with animal images taken at the same site using stones, trees, the horizon, or other landmarks visible within the image. In cases where these landmarks are not available, researchers may consider creating an artificial landmark, such as a strategically placed stone or log, which they can use to align their reference image and wildlife images with at the desktop. The reference image should contain the distance marker laid flat and out in front of the camera trap, directly in line with the lens. One should ensure that the camera trap used to take this reference image is in the same location and position as the camera trap used to collect wildlife images. The same model of camera trap must also be used. After this reference image is taken, the distance marker can be removed from the site.

### 2.4 Pixel measurements and conversion to centimeters

Images of the study species should feature the animal in clear view of the camera trap and within the range of the distance marker. Not all images need to be measured, as the sample size required will depend on the researcher's question and data availability. Representative images of the study species must be overlain with a reference image of the distance marker at the same site. This can be done using the transparency function in Photoshop, or with any equivalent image editing software that allows for image overlay and pixel measurements. To ensure accurate overlap, the reference image, and the wildlife image, should be aligned using objects in the background, such as trees, stones, or the edges of pathways (Figure 1). These objects can be traced to make this process easier (e.g., red drawn lines in Figure 1c). A wildlife image should only be used if the animal's whole flank is perpendicular to the orientation of the camera trap.

Using the “ruler” tool in the photo editing software, a straight, horizontal line is then drawn between the animal's front foot and the distance marker (Figure 1c). Where it overlaps on the distance marker is the distance of that animal to the camera trap. The ruler tool must be set to measure “pixels,” and then the height and length of the animal, or any other parameters of interest (e.g., head length, tail length, and flank width) can be measured. These measurements must be taken consistently (e.g., for height, starting from the front foot and measuring to the shoulder every time for each individual). The pixel measurements are converted to physical dimension measurements using the following equation, as per Leorna et al. (2022):

$$\text{Object size}\;\left(\mathrm{cm}\right)=\frac{\text{Object size}\;\left(\text{pixels}\right)\times \text{Distance to object}\;\left(\mathrm{cm}\right)}{\text{Focal length}\;\left(\text{pixels}\right)}$$

### 2.5 Test of accuracy in controlled conditions: Camera calibration

We calibrated camera focal length following the methods of Megalingam et al. (2016). We collected 15 images of a 25 cm × 25 cm piece of white paper on a poster board at 40‐cm intervals up to 200 cm distance. We took three images of the paper at each interval, ensuring the paper was at the center of the image and approximately perpendicular to the face of the camera trap. We measured the height and length of the paper in pixels using the ruler tool in Photoshop. We then estimated the focal length using the following equation:

$$d_{i}=\frac{s_{i}*d_{o}}{s_{o}}$$

From these estimates, we calculated the average focal length with 95% confidence intervals and employed the derived average focal length in all further equations.

### 2.6 Tests of accuracy

The accuracy of the pinhole camera method for estimating animal size has been effectively demonstrated by Leorna et al. (2022). However, as we are using a different model of camera trap and new method to derive distance of the animal to the camera trap, we validated the accuracy of our method in controlled conditions before undertaking our field measurements. To do this, we created silhouettes of *Felis catus* of four different sizes (Table 1). We collected one image of each silhouette at intervals of 20 cm between 110 and 210 cm from the camera trap. We also collected images of the silhouettes at unknown distances from the camera trap to determine how much additional error is incurred through the use of the portable reference marker. We measured height (front paw to shoulder) and length (base of tail to nose) for each silhouette and calculated percent relative error (RE) for each measurement (i.e., RE = ([estimated – actual]/actual) × 100). We calculated the average RE with 95% confidence intervals for images with known distance and images with unknown distances.

**TABLE 1:** Summary of results from accuracy trial of *Felis catus* silhouettes in controlled conditions.

Measured dimension	Known distance							Estimated distance						
	Relative error in estimated size				Actual error (cm)			Relative error in estimated size				Actual error (cm)		
	*n*	Mean	95% CI		Mean	95% CI		*n*	Mean	95% CI		Mean	95% CI	
			LL	UL		LL	UL			LL	UL		LL	UL
Height	24	−0.88%	−1.71	−0.04	−0.21	−0.4	−0.02	32	5.06%	2.91	7.2	1.23	0.73	1.75
Length	24	−0.49%	−0.99	0.03	−0.3	−0.57	−0.02	32	6.61%	4.54	8.66	3.4	2.31	4.56

*Note*: Confidence intervals (CI) are reported with lower limits (LL) and upper limits (UL).

### 2.7 Application of methodology using feral cats as a case study

The physiology of *F. catus*, or the domestic cat, is well studied in the context of veterinary science (Courchamp et al., 2000), but understudied within the feral populations of Australia, with feral cats defined here as “cat that lives in the wild and can survive without human reliance or contact” (Garrard et al., 2020). There are many anecdotal reports of large cats published within social and popular media (Menkhorst & Morison, 2012), but limited published empirical evidence on their body size in an Australian context. Feral cat size may also have some relevance to the threat posed by feral cats to Australian wildlife, as it has been suggested that as feral cats get bigger, they consume a greater quantity and more diverse prey items (Yip et al., 2015).

Camera traps have been revolutionary for monitoring feral cats (Bengsen et al., 2012) and could be used to provide information on cat size. In this case study we estimate feral cat size with camera trap images using data collected in a temperate rainforest/wet‐eucalypt forest, an environment where the shooting and trapping of live cats is infeasible. The frequent capture and re‐capture of feral cats within a pre‐existing camera trap survey in the south‐east of Tasmania provided a large dataset for us to test and demonstrate the utility of our size methodology, while also giving us the opportunity to evaluate the size of individuals within this wild population.

### 2.8 Existing field sites and camera deployment

**FIGURE 2:**
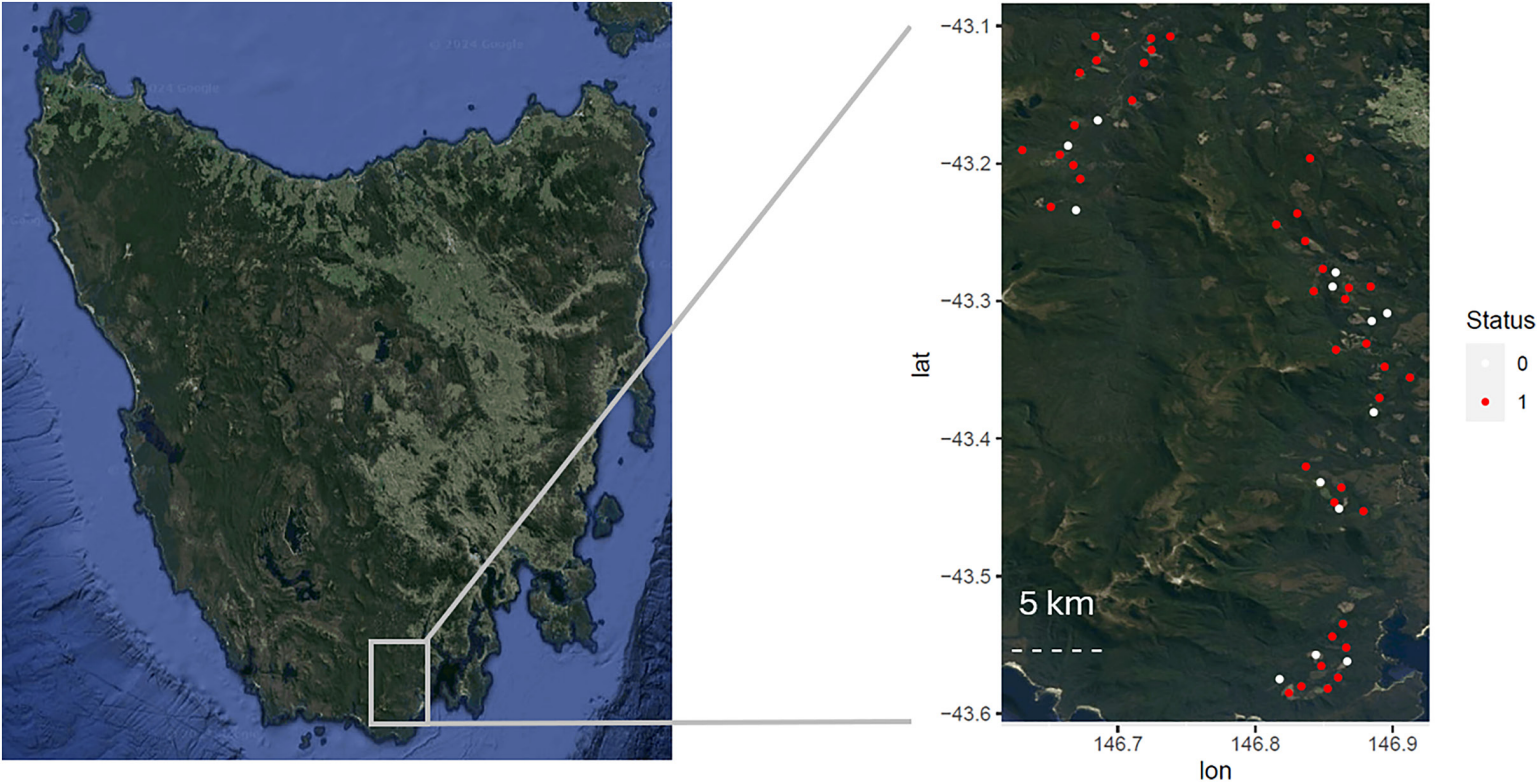
Map of Tasmania showing the camera trap sites used in this study. Status indicates whether a site provided measurable data, with red points (1) as sites that provided data suitable for size measurements of cats, and white (0) with no useable photos.

We reviewed images from an existing dataset of 54 trail cameras (model: Cuddeback X‐Change 1279) that were deployed in the Picton region of Tasmania as a part of a broader camera trap network (B.W. Brook and J.C. Buettel unpublished) (Figure 2). Cameras were secured to trees at animal shoulder height, 30–50 cm, as per Apps and McNutt (2018). Of the 54 cameras, 48 were set on unsealed forestry roads and six off the road in nearby natural arenas or on game trails, and no lures were used. All cameras used a white flash with a passive infra‐red sensor (being triggered in response to movement and heat), and a minimum lapse time between successive of 30 s during the day and 1 min at night. Considering that all camera traps were deployed more than 5 km from the nearest town (population 125 people), all cats observed were assumed to be feral.

### 2.9 Processing camera trap images

Reference images were taken at all 54 camera sites during the final service in 2021 using the methodology as described above (see section: *Distance marker and in‐field protocol*). Images for size estimation were included only if the cat was within 2.3 m of the camera and had their flank parallel to the camera trap. Of the 54 sites, 32 (60%) met these criteria, having images of cats close enough to the camera trap to reliably obtain distance to the camera, and where cats were at an appropriate angle to be measured accurately. From the chosen sites, we had access to 327 images of cats for measurement. Of these, 157 featured black cats, and 170 photos could be identified at the individual level.

Although our method does not require individual identification for size estimation, employing it enables us to scrutinize the standard errors attributed to each individual and thereby assess the precision of our methodology. We identified individual cats by their unique coat markings where possible, excluding black cats from our analysis. To mitigate potential bias from site‐specific camera characteristics—such as road width and camera angle—a maximum of 10 images per individual cat were processed for each camera. If only a single image was captured for an individual across all sites, then this individual was excluded from our analysis and method demonstration. Using this protocol, 32 unique individual cats were identified and measured. Cat height (front paw to shoulder) and length (base of tail to nose) were measured in pixels. To provide an indication of precision, measurements for each individual cat was averaged and confidence intervals were calculated using the bootstrap method in R (Canty & Ripley, 2017).

## 3 RESULTS

### 3.1 Camera calibration and accuracy

The mean derived focal length for the Cuddeback X‐Change 1279 model with an image resolution of 20mp was 6747.9px (95% CI = 6711.5, 6784.2). The mean RE for estimates of height and length for measurements taken with known distance to camera trap were − 0.88% (CI = −1.71, −0.04) and −0.49% (CI = −0.99, 0.03), respectively (Table 1). The mean RE for estimates of height and length with estimated distance to camera trap were 5.06% (CI = 2.91, 7.2) and 6.61% (CI = 4.54, 8.66), respectively (Table 1). Measurements of individual silhouettes and their RE are reported in Table S1.

### 3.2 Field trial results: Feral cat size estimates

**FIGURE 3:**
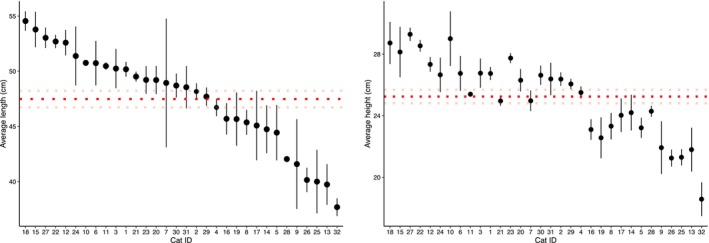
Comparisons of average cat length (left) and height (right) for each individual cat (labelled 1–32) with standard error bars. Note that the x‐axis is in descending order for length, and that the cat IDs on the x‐axis for average height match that order to allow for comparisons between individuals. The dashed red line in each graph displays the average length and height across all individuals, and the faint dashed red lines above and below this show the average +/− one standard error.

**FIGURE 4:**
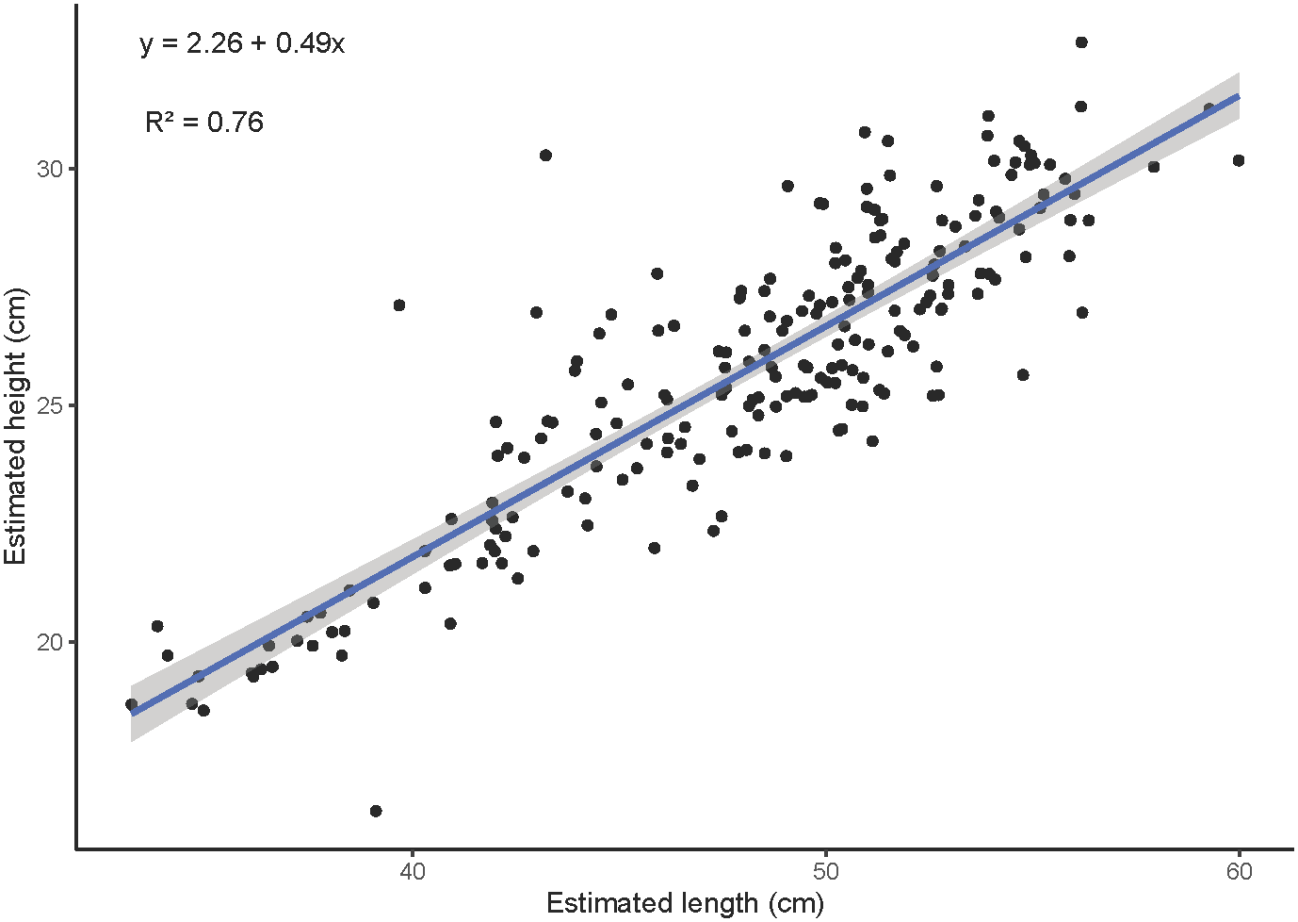
Relationship between estimated length and height (centimeters) within each image. Points represent each image measured, with the regression line provided in blue and standard error in gray.

Average cat height for the population was 25.25 cm (CI = 24.4, 26.1) and the average length was 47.48 cm (CI = 46.0, 48.9) across all measured cat images. The average standard error for each individual was 1.58 cm for height (CI = 1.2, 2.0) and 0.82 cm for length (CI = 0.66, 0.97). The tallest individual's average height was 29.3 cm (CI = 28.0, 30.5), and the longest individual's average length was 54.6 cm (CI = 49.0, 60.1). The shortest individual had an average height of 18.6 cm (CI = 15.4, 21.8), and the individual with the shortest length measured 37.7 cm (CI = 35.3, 40.0) (Figure 3). There was a strong relationship between height and length across all measured images (*R* ^2^ = .87) (Figure 4).

## 4 DISCUSSION

Our initial tests of accuracy under controlled conditions showed that our novel method to derive animal distance to the camera trap resulted in a consistent overestimation of animal size of around 2%–8%. While greater than our relative error when distance was known, this margin of error compares favorably with past studies utilizing the pinhole approach (Leorna et al., 2022). As such, we were able to confidently derive consistent estimates of height and length for 32 unique cats as calibrated against repeated images. Notably, our measurements were close to the expected range of body size for domestic *F. catus* globally: 46 cm for length and 23–25 cm for height (Sunquist, 2002). The key advantages of our methodology for measuring body size in camera trap images are two‐fold: (i) the distance marker does not need to remain at the field‐site, thus lowering costs and mitigating theft risk, and (ii) as a consequence, this approach can be used to measure animal body size from historical datasets, by re‐visiting the site and replicating the position of the previously placed camera, and taking a reference image with the distance marker in place.

We were able to integrate our method of estimating body size into a pre‐existing camera trap survey as our camera traps were still operational in the field. For researchers wishing to apply this method to a historical dataset, sufficient information must be available regarding the camera trap's location and positioning. This includes the height of the camera trap, angle, and tree/location of the post on which the camera was deployed. It is recommended that the height and angle of the camera trap are reported in all camera trap studies, along with habitat information (Meek et al., 2014). Researchers who have photographed their camera trap deployments when recording this information will likely find it easier to apply our method to their historical datasets.

**FIGURE 5:**
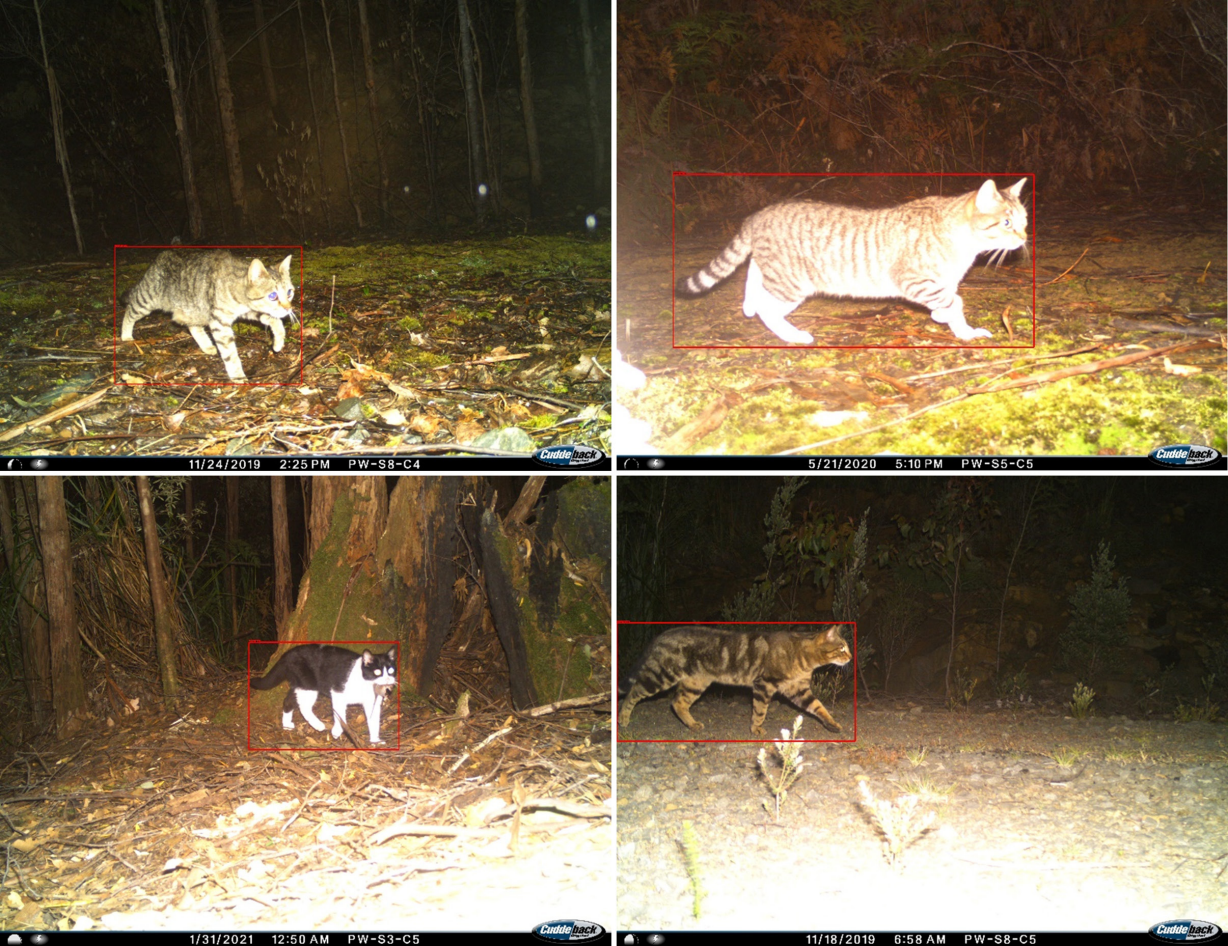
On the left, two examples of cats on an angle, making them inappropriate to measure for dimensions like length, which require a full view of the animal's flank, from these camera trap images. On the right, two examples of cats that are parallel and close to the camera trap, making them good specimens for measurement using these camera trap images.

The margin of error varied among individuals: some exhibited consistent size estimates across images, while others showed greater variability. This variability could be attributed to the differences in the position of the animal in each photograph (Tarugara et al., 2019), or some foreshortening introduced by slightly oblique angles (e.g., Figure 5, left panels). This corroborates the findings of Leorna et al. (2022), who noted a decline in measurement accuracy for reindeer as the distance to the camera trap increased or when photographed at an angle. Additional variation in our measurements was also introduced by the non‐exact nature of our distance marker, which measured distance in intervals of 10 cm. This is because the resolution of the camera trap images was too low to examine more precise intervals (e.g., 5 cm and 1 cm). Despite these potential sources of error, the confidence intervals were tight for most individuals, indicating that our method provided consistent measurements despite uncertainties in angles and distance among camera sites. Obtaining pixel measurements for each image was done by hand within out study, although the utilization of deep‐learning image processing software by future researchers could provide an automated alternative. For example, technology in aquaculture has been developed to automatically measure fish snout‐to‐fork length in pixels (Tseng et al., 2020), and while an initial set of training data would need to be provided to facilitate a similar approach here, this approach could reduce some of the labor involved with our current method.

In our case study, camera traps placed off‐trail captured fewer cats than road cameras, and these cats were often approaching the camera or walking away from it, making the images unmeasurable. Lures have been used in past studies to overcome this problem, increasing capture rate and to ensure the animal is perpendicular to the camera at the time of capture so that dimensions can be measured (Tarugara et al., 2019). Our study shows that roads and trails can be used in the same capacity. Predators have high rates of detection on roads and trails (Wysong et al., 2020), making these locations a sort of “passive lure” for feral cats. In addition, we note that the road locations measured in our study consistently yielded images of cats positioned with their flanks parallel to the camera trap, as they were following a directed linear path of movement (Figure 5), and these animals were also generally close to the camera trap (i.e., within 230 cm). Our portable distance marker is advantageous in this context, as permanent distance markers cannot be placed on active roads and may increase the risk of theft on walking tracks by making the camera trap's location more obvious to human observers. This was a particular concern in Tasmania, where 20% of camera traps from the broader monitoring network were stolen over a four‐year study period (L.M Cardona, B.W. Brook, Z. Aandahl, and J.C. Buettel, unpublished). As such, our distance marker methodology can be applied to predator surveys already utilizing these locations of high predator traffic where distance markers have previously been unsuitable.

Our field trial provided the first estimates of the size of feral cats living in the dense rainforests and tall wet forests of south‐east Tasmania, in areas remote from human settlements. The average estimates of height and length for cats in our study was 25.3 cm (CI = 24.4, 26.1) and 47.5 cm (CI = 46.0, 48.9), which is similar to that of typical domestic cats of 46 cm for length and 23–25 cm for height (Sunquist, 2002). This is particularly true when one considers that our accuracy trial indicated that our methodology consistently over‐estimates dimensions. As such, our findings do not contain any evidence that supports the phenomenon of Australian “panthers” and “big cats,” which is commonly reported in the media, but not currently supported by any scientific literature (Menkhorst & Morison, 2012). However, we have only sampled a small pocket of the Tasmanian wilderness herein. The pinhole camera approach we employed provides an opportunity to substantiate claims of giant cats in Australia where shooting and trapping fail or are unavailable, particularly considering this method can be applied to historic data.

## 5 CONCLUSION

The pinhole camera approach is a cost‐effective method to estimate animal body size if using pre‐existing camera trap surveys, allowing researchers to exploit past data. There are several caveats associated with our method. A researcher must return to the site of a camera trap survey and replicate deployment to obtain a reference image if using past data, and a wildlife image should only be used if the animal photographed is parallel to the camera‐trap, such that the whole flank can be seen. This can limit the amount of data available to be used. Additionally, pixel measurements need to be taken consistently, although this step could be aided by the integration of a measurement AI. Nonetheless, this method provides a non‐invasive alternative to live capture or killing that consistently provides precise animal dimensions. We encourage other researchers to test the pinhole approach with other models of camera trap, species, or captive populations to further validate this method.

## AUTHOR CONTRIBUTIONS

**Alexandra J. Paton:** Conceptualization (lead); data curation (equal); formal analysis (lead); investigation (lead); methodology (lead); resources (supporting); visualization (lead); writing – original draft (lead); writing – review and editing (equal). **Barry W. Brook:** Conceptualization (supporting); data curation (equal); supervision (equal). **Jessie C. Buettel:** Conceptualization (supporting); data curation (equal); supervision (lead); writing – review and editing (lead).

## FUNDING INFORMATION

Funding provided by the Australian Research Council (FL160100101).

## CONFLICT OF INTEREST STATEMENT

The authors declare no conflict of interest.

## Supporting information


Table S1

## Data Availability

All data and code used are available on GitHub: https://github.com/AlexJPaton/BodyDimensions.
