# Supplementary material for: A non‐invasive approach to measuring body dimensions of wildlife with camera traps: A felid field trial
Source: Ecol Evol. 2024 Jul 1;14(7):e11612. doi: 10.1002/ece3.11612 (PMC11216788; doi:10.1002/ece3.11612)
Supplement: Supplementary file 1 — Table S1 [file ECE3-14-e11612-s001.docx]

Table S1: Precision and accuracy of estimated Felis catus dimensions from silhouettes of known size. Mean, upper 95% confidence intervals (UL), and lower 95% confidence intervals (LL) are provided in centimetres. Relative error (RE) and its standard error (SE) are provided as percentages.

|  | | | | **Estimated length** | | | | | |  | | **Estimated height** | | | | |
| --- | --- | --- | --- | --- | --- | --- | --- | --- | --- | --- | --- | --- | --- | --- | --- | --- |
| **Trial** | **Individual** | ***n*** | **Actual length** | **Mean** | **LL** | **UL** | **RE** | **SE** | **Actual height** | | **Mean** | | **LL** | **UL** | **RE** | **SE** |
| Known distance to camera | 1 | 6 | 43 cm | 43.1 | 42.6 | 43.7 | 0.3 | 0.6 | 22.5 cm | | 22.0 | | 21.6 | 22.4 | -2.3 | 0.9 |
|  | 2 | 6 | 48 cm | 47.9 | 47.3 | 48.4 | -0.3 | 0.6 | 23 cm | | 23.1 | | 22.7 | 23.5 | 0.4 | 0.9 |
|  | 3 | 6 | 56.5 cm | 56.0 | 55.5 | 56.5 | -0.9 | 0.4 | 25 cm | | 24.9 | | 24.5 | 25.2 | -0.6 | 0.8 |
|  | 4 | 6 | 64 cm | 63.3 | 62.7 | 63.8 | -1.1 | 0.4 | 27 cm | | 26.7 | | 26.3 | 27.1 | -1.1 | 0.7 |
| Estimated distance to camera | 1 | 8 | 43 cm | 46.3 | 44.4 | 48.2 | 7.6 | 2.3 | 22.5 cm | | 23.3 | | 22.4 | 24.3 | 3.7 | 2.2 |
|  | 2 | 8 | 48 cm | 51.4 | 49.3 | 53.5 | 7.0 | 2.3 | 23 cm | | 24.5 | | 23.5 | 25.5 | 6.4 | 2.3 |
|  | 3 | 8 | 56.5 cm | 60.0 | 57.5 | 62.5 | 6.2 | 2.3 | 25 cm | | 26.3 | | 25.3 | 27.4 | 5.3 | 2.2 |
|  | 4 | 8 | 64 cm | 67.6 | 64.8 | 70.4 | 5.6 | 2.3 | 27 cm | | 28.3 | | 27.2 | 29.4 | 4.9 | 2.2 |
